# Supplementary material for: How Will I Evaluate Others? The Influence of “Versailles Literature” Language Style on Social Media on Consumer Attitudes Towards Evaluating Green Consumption Behavior
Source: Behav Sci (Basel). 2025 Jul 17;15(7):968. doi: 10.3390/bs15070968 (PMC12292707; doi:10.3390/bs15070968)
Supplement: Supplementary file 1 [file behavsci-15-00968-s001.zip › behavsci-3664436-supplementary.pdf]

Table S1. Validity Analysis Validity Analysis of Experiment 1

variables	CR	AVE	BP	HP	LS
BP	0.948	0.860	0.927		
HP	0.963	0.897	0.887***	0.948	
LS	0.944	0.851	-0.662***	-0.641***	0.923

Table S2. HTMT Analysis of Experiment 1

variables	BP	HP	LS
BP			
HP	0.892		
LS	0.655	0.631	

Table S3. Mann-Whitney U for mediating variables and dependent variablesof Experiment 1

non-Versailles Literature and Versailles Literature	Z	P
bragging perception	-5.483	0.000
hypocrisy perception	-5.888	0.000
attitudes toward the poster's green consumption behavior	-4.637	0.000

Table S4. Validity Analysis Validity Analysis of Experiment 2

variables	CR	AVE	BP	HP	LS
BP	0.923	0.800	0.894		
HP	0.951	0.866	0.884***	0.931	
LS	0.955	0.876	-0.833***	-0.894***	0.935

Table S5. HTMT Analysis of Experiment 2

variables	BP	HP	LS
BP			
HP	0.884		
LS	0.833	0.893	

Table S6. Mann-Whitney U for mediating variables and dependent variables of Experiment 2

non-Versailles Literature acquaintances and stranger Group	Z	P	Versailles Literature acquaintances and stranger Group	Z	P
bragging perception	-2.168	0.030	bragging perception	-4.040	0.000
hypocrisy perception	-0.832	0.406	hypocrisy perception	-3.951	0.000
attitudes toward the poster's green consumption behavior	-0.842	0.400	attitudes toward the poster's green consumption behavior	-3.927	0.000

Table S7. Mann-Whitney U for mediating variables and dependent variables of Experiment 3

non-Versailles Literature acquaintances and stranger Group	Z	P	Versailles Literature acquaintances and stranger Group	Z	P
bragging perception	-0.195	0.845	bragging perception	-3.211	0.001
hypocrisy perception	-0.082	0.935	hypocrisy perception	-4.314	0.000
attitudes toward the poster’s green consumption behavior	-0.504	0.614	attitudes toward the poster’s green consumption behavior	-4.526	0.000
